# Rapid influenza molecular testing in secondary care and influenza surveillance in England: Any impact?

**DOI:** 10.1111/irv.13001

**Published:** 2022-05-18

**Authors:** Nicki L. Boddington, Suzanne Elgohari, Joanna Ellis, Matthew Donati, Maria Zambon, Richard G. Pebody

**Affiliations:** ^1^ Immunisation and Vaccine‐Preventable Diseases Division UK Health Security Agency London UK; ^2^ South West Regional Laboratory and Severn Infection Sciences North Bristol NHS Trust Bristol UK; ^3^ Reference Microbiology Services WHO Regional Office for Europe Copenhagen Denmark

**Keywords:** influenza surveillance, rapid influenza testing, severe influenza

## Abstract

**Introduction:**

The use of rapid molecular testing for influenza diagnosis is becoming increasingly popular. Used at the point of care or in a clinical laboratory, these tests detect influenza A and B viruses, though many do not distinguish between influenza A subtypes. The UK Severe Influenza Surveillance System (USISS) collects surveillance data on laboratory‐confirmed influenza admissions to secondary care in England.

This study set out to understand how rapid influenza molecular testing was being used and how it might influence the availability of subtyping data collected on influenza cases admitted to secondary care in England.

**Methods:**

At the end of the 2017/2018 and 2018/2019 influenza seasons, a questionnaire was sent to all National Health Service Hospital Trusts in England to evaluate the use of rapid influenza testing. Surveillance data collected through USISS was analysed from 2011/2012 to 2020/2021.

**Results:**

Of responding trusts, 42% (13/31) in 2017/2018 and 55% (9/17) in 2018/2019 used rapid influenza molecular tests, either alone or in combination with other testing. The majority of rapid tests used did not subtype the influenza A result, and limited follow‐up testing occurred.

Surveillance data showed significant proportions of influenza A hospital and intensive care unit/high dependency unit admissions without subtyping information, increasing by approximately 35% between 2012/2013 and 2020/2021.

**Conclusions:**

The use of rapid influenza molecular tests is a likely contributing factor to the large proportion of influenza A hospitalisations in England that were unsubtyped. Given their clear clinical advantages, further work must be done to reinforce these data for public health through integrated genomic surveillance.

## 1 INTRODUCTION

The use of rapid molecular testing methods for influenza diagnosis is becoming increasingly popular to help in the management of patients who present with symptoms compatible with influenza. Such methods used at the point of care (POC), that is, at the bedside, or in a clinical laboratory, can provide results in a relatively short and clinically relevant time period, often in less than 60–90 min,^1^, ^2^, ^3^ and faster than conventional techniques.^4^ Early diagnosis of influenza can facilitate early initiation of antiviral treatment and rapidly guide implementation of recommended infection and prevention control measures.^4^, ^5^

These tests are most commonly nucleic acid amplification based (NAAT) and detect influenza A and B viruses, though not all have the capability for subtyping of seasonal influenza A type viruses. These rapid NAATs have improved sensitivities compared to rapid antigen detection tests, and for some rapid NAATs, this is similar to those of real‐time polymerase chain reaction (RT‐PCR) assays.^3^, ^4^ The use of rapid influenza testing may, however, have potential negative implications for traditional epidemiological surveillance including the capturing of results, the reduced availability of influenza A subtyping information, and the referral of samples for onward testing including virus characterisation.

The UK Severe Influenza Surveillance System (USISS) was established following the 2009 pandemic to monitor severe seasonal influenza.^6^ The system collects surveillance data on laboratory confirmed influenza admissions to secondary care in England through two schemes: the mandatory and the sentinel schemes (Scheme 7). The system aims to monitor the impact of influenza on the population and describe the epidemiology of severe influenza in time, place, and person.^7^ The impact of the use of rapid influenza testing on the surveillance of severe influenza collected by this scheme is currently unknown.

The purpose of this study was to evaluate the use of rapid testing in secondary care in England and to understand how its use might influence the availability of subtyping data collected on influenza cases admitted to secondary care in England.

## 2 METHODS

The USISS Mandatory and Sentinel hospital networks have operated fully since the 2011/2012 influenza season. The USISS Mandatory scheme is a mandatory collection of the weekly number of laboratory confirmed influenza admissions to intensive care units (ICUs) and high dependency units (HDUs) by age group and influenza type and subtype from all National Health Service (NHS) Hospital Trusts in England. The Sentinel scheme collects the weekly number of laboratory confirmed influenza admissions at all levels of care, by age group and influenza type and subtype from a sentinel network of NHS Hospital Trusts in England. The recruitment of NHS Hospital Trusts into the Sentinel system has been described elsewhere.^8^

A laboratory‐confirmed hospitalised case was defined as any person who was hospitalised and had laboratory‐confirmed influenza A (A(H1N1)pdm09, A(H3N2) or subtype unknown) or influenza B infection. For the purposes of ICU/HDU surveillance, a confirmed case was defined as any person who was admitted to ICU/HDU and had laboratory‐confirmed influenza A (A(H1N1pdm09), A(H3N2) or subtype unknown) or influenza B infection.

During the study period (2011/2012 to 2020/2021), both collections ran from the beginning of October (week 40) to mid‐May (week 20) and were web based, with NHS Hospital Trusts reporting data through online web portals. The legal basis for the USISS Mandatory scheme is sections 254(1) and (6); 260(2)(d); 261(3); and 304(9), (10) and (12) of the Health and Social Care Act.^9^

At the end of the 2017/2018 and 2018/2019 influenza seasons, a questionnaire was sent to all participating NHS Hospital Trusts. The questionnaire included questions on the types of diagnostic testing for influenza used in Trusts and, in particular, their use of rapid influenza molecular tests (i.e. those with results in a short and clinically relevant time period). The 2018/2019 questionnaire was extended to include questions on the capability of the rapid influenza tests to provide subtyping information along with follow up questions regarding the referral of samples for follow up testing and the reporting of these results for trusts using rapid tests that did not provide subtyping information.

In 2017/2018, the survey was emailed to all participating Mandatory trusts as a Microsoft Word document, and Trusts were asked to return it by email. In 2018/2019, the survey was administered via an online survey tool, the link for which was emailed to all participating trusts (from the mandatory and sentinel schemes). Emails in both seasons were sent to known contacts from the participating trusts, often those with responsibility for submitting data to the schemes during the season, and reminder emails were sent to optimise response rates.

## 3 RESULTS

### 3.1 Survey results

In 2017/2018, the survey was sent to 144 NHS Hospital Trusts participating in the Mandatory USISS scheme, and in 2018/2019, the survey was sent to 143 NHS Hospital Trusts (including to contacts from both the Mandatory and Sentinel trusts). The response rate for both seasons was 22% (*n* = 31) with representation from all regions and trust types in England. Fifteen trusts completed the surveys in both seasons. Trust size (number of beds) in participating trusts ranged from 143 to 2000 in both seasons with a median of 857 in 2017/2018 and 787 beds in 2018/2019. In 2018/2019, the median number of patients tested for influenza in all settings (including Accident and Emergency, hospital wards and ICU/HDU) was 1751 with a range of 142 to 5790 patients per trust, among the 23 trusts who responded to this question.

In 2017/2018, rapid influenza tests used in any setting (i.e. clinical settings and/or in the laboratory), whether alone or in combination with routine laboratory testing, were used in 42% (13/31) of responding trusts. The 39% of responding trusts (12/31) reported using only multiple patient, batched/routine testing in local/host laboratory, and the remaining 19% (6/31) reported using multiple patient batched/routine testing in another laboratory/outside host trust. In 2018/2019, rapid influenza tests used in any setting, whether alone or in combination with routine laboratory testing, had increased to 55% (17/31) of responding trusts. 23% of responding trusts (7/31) reported using batched/routine testing in local/host laboratory only, and 16% (5/31) reported using batched/routine testing in another laboratory/outside host trust. The remaining 6% reported using either a combination of multiple patient batched/routine testing in local or non‐local laboratory or other test type.

In 2018/2019, of those responding trusts who used rapid influenza tests (whether alone or in combination with other routine laboratory testing), 53% (9/17) said that the type of rapid test used did not subtype the samples tested. Of these trusts, 56% (5/9) said that no follow up testing/confirmation/subtyping was carried out on these samples.

In the 2017/2018, 29% (9/31) of trusts stated they had plans to increase rapid influenza testing in the 2018/2019 influenza season. By 2018/2019, this has increased to 48% of trusts reporting an increase in rapid influenza testing year on year within their trust (15/31) with 48% also stating that the trust has plans to increase rapid influenza testing for the forthcoming influenza season (15/31).

### 3.2 Surveillance results

**FIGURE 1:**
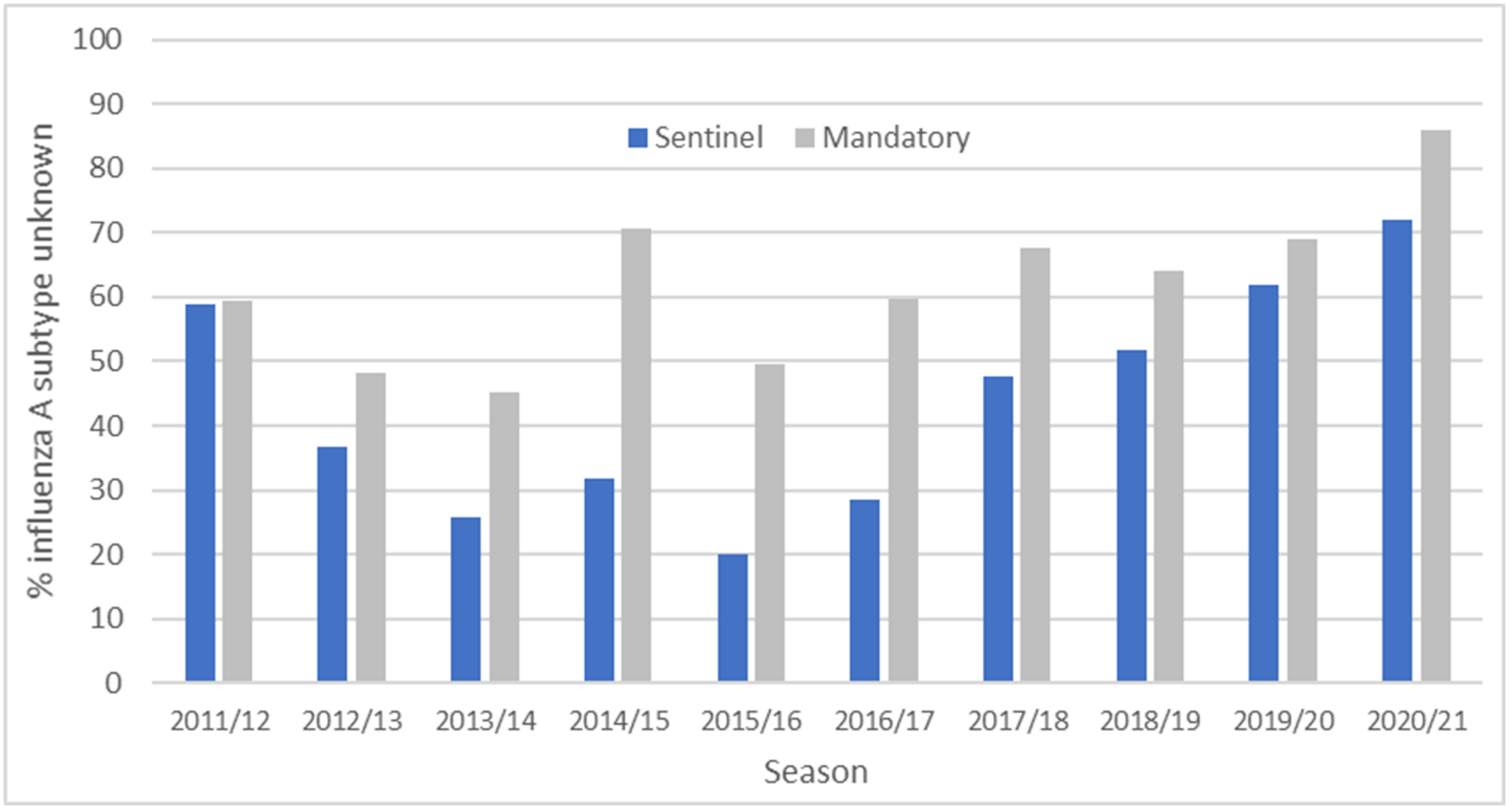
Proportion of influenza A results with unknown subtype by influenza season and surveillance system, 2011/2012 to 2020/21

Surveillance data from 2011/2012 to 2020/2021 showed varying proportions of laboratory confirmed hospitalisations and ICU/HDU admissions without influenza A subtyping information. The proportion of influenza A admissions with unknown subtype have been increasing through both USISS schemes in recent seasons (Figure 1). Generally, a greater proportion of influenza A ICU/HDU admissions (reported through the mandatory scheme) had missing subtype information compared with influenza A hospitalisations (reported through the sentinel scheme) (Figure 1).

The first year of the operation of the systems was 2011/2012. From 2012/2013, when the system was embedded, the proportion of influenza A ICU/HDU admissions with unknown subtype increased from 48.3% to 86.0% in 2020/2021, and the proportion of influenza A hospitalisations with unknown subtype increased from 36.7% to 72.0% in the sentinel system (Figure 1).

## 4 CONCLUSIONS

Data collected through the hospital‐based surveillance system, USISS, since its conception has allowed for inter‐seasonal comparisons of influenza and has provided a unique opportunity to describe the epidemiology of severe influenza in England. This study has demonstrated, through the results of a survey sent to participating USISS trusts in England over two influenza seasons, that the use of rapid influenza molecular testing, either alone or in combination with other testing, is now prevalent amongst NHS Hospital Trusts in England. Furthermore, the majority of Trusts reported an increase in rapid testing over two seasons within their Trusts with the majority planning to continue increasing their use in the subsequent influenza season. This increase coincides with an increase in the proportion of unsubtyped influenza A reports from the hospital and ICU reporting systems. Large overall increases were observed over the study period with high proportions of unsubtyped influenza A reports from both schemes by the end of the study period. This is despite low influenza activity in the last two seasons due to the COVID‐19 pandemic. In Europe, a similar picture of lack of influenza A subtyping for laboratory confirmed cases in ICU can be seen in this current influenza season.^10^

The survey also highlighted that when rapid influenza testing was used, just over half of the tests used did not subtype the influenza A positive samples and few of these samples were referred for any follow‐up testing or confirmation. Of those samples that were referred for follow‐up testing, it is unknown to what extent confirmatory results were received back and whether hospital Laboratory Information Management Systems (LIMS) were updated. Lack of subtype information affects the accuracy of epidemiological analysis of the impact of different influenza subtypes on disease burden, epidemic thresholds for defining severity, and may affect the parameterisation of dynamic models that may be used to assess influenza transmission.

Such challenges of rapid tests from a surveillance perspective have been documented elsewhere.^11^, ^12^ In particular, rapid tests were introduced in Scotland in 2017/2018 when moderate to high levels of influenza activity were putting pressure on bed occupancy within the hospital system. Whilst this was found to have a positive impact on local bed occupancy and treatment and infection control measures, due to a lack of provision to enable the results of the rapid tests to be captured by the Scottish national surveillance system, there was a loss of data to the national surveillance system.^12^ In the 2018/2019 season, most positive results were believed to be captured by the national system; however, the same was not thought to be true of negative results, with a sizable proportion of negative results not being captured by the national system.^12^

This study is limited by the low response rates to the surveys. However, the sampling frame was large, leading to a reasonable number of sampled sites for the purposes of this study. A better response rate may have enabled questions around the reporting of subtyping results from referred samples and potential for duplication of reporting to be answered, along with more information about the location of patients when respiratory samples were collected for rapid influenza testing such as Emergency Departments, hospital wards or ICU/HDU, and the type of respiratory specimens collected such as upper respiratory tract only, or both upper and lower respiratory tract specimens. Another possible limitation is the lack of anonymisation of the survey which may have meant that trusts hesitant to admit practice might have been less likely to complete the survey.

This increase in use of rapid influenza testing poses a number of possible benefits as well as some challenges from a surveillance perspective (aside from the potential clinical and hospital management advantages). Notably, the increase in rapid testing may result in a more complete and timely picture of the epidemiology of severe influenza in England. In the United States (US), a retrospective study demonstrated that POC testing had the potential to generate an early alert signal before the epidemic onset of influenza in the community and ahead of traditional surveillance methods.^13^ However, a key challenge that is posed by rapid influenza testing is the potential for an increase in the number of samples from severe cases that remain unsubtyped. The USISS data included in this study have demonstrated that a significant proportion of influenza A admissions remain unsubtyped on an annual basis, and this proportion has gradually increased in the most recent seasons, including those where there was limited influenza activity due to the COVID‐19 pandemic. Despite this, the increasing trend has not been entirely progressive over time and differed slightly between the two reporting schemes. Several reasons may have influenced these trends including changes in diagnostic assays, practice of reporting trusts such as capacity issues, updating of results once a subtyping result was available and the evolution of LIMS systems over time. The COVID‐19 pandemic is likely to have impacted the testing landscape further for influenza with hospitals now routinely using rapid POC tests to detect SARS‐CoV‐2 as well as the development of multiplex RT‐PCR assays and rapid antigen tests. Since hospitals are now routinely using rapid POC tests for COVID‐19, they may be more likely to expand the use of rapid testing to include influenza, and the development of tests that can distinguish between SARS‐CoV‐2 and influenza may increase this likelihood further. An additional limitation of the use of rapid tests for secondary care surveillance is the potential for false‐negative results given that progression to severe disease can occur late in illness when rapid tests may lack the sensitivity to be able to detect infection.

The clinical advantages of rapid tests in acute hospital settings are clear, potentially enabling patients to be more rapidly triaged, opportunities for nosocomial transmission reduced and timely clinical management interventions to be implemented,^14^, ^15^, ^16^ and we have demonstrated increasing use of these tests within secondary care in England. We have also demonstrated a large proportion of influenza A surveillance reports for which subtyping information is not available. The increasing adoption of rapid tests is likely to be an important explanatory factor. The need for further sampling or additional work on samples may be reducing specimen referrals for subtyping and affecting data flows for national surveillance. Hospital trusts in England should continue to be encouraged to ensure that all test results are integrated into local LIMS systems and that subtyping is performed locally, where possible, or regionally. Reinforcing the importance of contributing samples and data for public health surveillance is the key for forming a more complete picture of circulating influenza subtypes and strains associated with severe outcomes alongside the development and use of rapid tests that have the ability to identify influenza A virus subtypes.

## AUTHOR CONTRIBUTIONS

**Nicki Boddington:** Data curation; formal analysis; project administration. **Suzanne Elgohari:** Data curation; formal analysis. **Joanna Ellis:** Data curation. **Matthew Donati:** Data curation. **Maria Zambon:** Conceptualization; data curation. **Richard Pebody:** Conceptualization; data curation; methodology; supervision.

## FUNDING STATEMENT

None.

### PEER REVIEW

The peer review history for this article is available at https://publons.com/publon/10.1111/irv.13001.

## Data Availability

The data that support the findings of this study are available from the corresponding author upon reasonable request.

## References

[1] Centers for Disease Prevention and Control . Information on rapid molecular assays, RT‐PCR, and other molecular assays for diagnosis of influenza virus infection 2019 [Available from: https://www.cdc.gov/flu/professionals/diagnosis/molecular-assays.htm.]

[2] Public Health England . Point of care tests for influenza and other respiratory viruses 2018 [Available from: https://www.gov.uk/government/publications/point-of-care-tests-for-influenza-and-other-respiratory-viruses.]

[3] Azar MM , Landry ML . Detection of influenza A and B viruses and respiratory syncytial virus by use of Clinical Laboratory Improvement Amendments of 1988 (CLIA)‐waived point‐of‐care assays: a paradigm shift to molecular tests. J Clin Microbiol. 2018;56(7). doi:10.1128/JCM.00367-18 PMC6018333 29695519

[4] Vos LM , Bruning AHL , Reitsma JB , et al. Rapid molecular tests for influenza, respiratory syncytial virus, and other respiratory viruses: a systematic review of diagnostic accuracy and clinical impact studies. Clin Infect Dis. 2019;13;69(7):1243‐1253. doi:10.1093/cid/ciz056 PMC7108200 30689772

[5] Egilmezer E , Walker GJ , Bakthavathsalam P , et al. Systematic review of the impact of point‐of‐care testing for influenza on the outcomes of patients with acute respiratory tract infection. Rev Med Virol. 2018;28(5):e1995. doi:10.1002/rmv.1995 30101552 PMC7169080

[6] Bolotin S , Pebody R , White PJ , McMenamin J , Perera L , Nguyen‐Van‐Tam JS , Barlow T , Watson JM , for the UK Severe Influenza Surveillance System (USISS) Steering Group . A new sentinel surveillance system for severe influenza in England shows a shift in age distribution of hospitalised cases in the post‐pandemic period. PloS One 2012;7(1):e30279. doi:10.1371/journal.pone.0030279 22291929 PMC3264602

[7] Boddington NL , Verlander NQ , Pebody RG . Developing a system to estimate the severity of influenza infection in England: findings from a hospital‐based surveillance system between 2010/2011 and 2014/2015. Epidemiol Infect. 2017;145(7):1461‐1470. doi:10.1017/S095026881700005X 28166855 PMC9203293

[8] World Health Organization Regional Office for Europe and European Centers for Disease Control and Prevention . Flu News Europe ‐ Joint ECDC‐WHO/Europe Weekly Influenza Update ‐ Hospital Surveillance 2022 [Available from: https://flunewseurope.org/HospitalData.]

[9] Health and Social Care Act 2012 [Available from: http://www.legislation.gov.uk/ukpga/2012/7/contents/enacted]

[10] Dickson EM , Zambon M , Pebody R , et al. Do point‐of‐care tests (POCTs) offer a new paradigm for the management of patients with influenza? EuroSurveillance. 2020;25(44). doi:10.2807/1560-7917.ES.2020.25.44.1900420 PMC7645971 33153522

[11] Dickson EM , Marques DF , Currie S , et al. The experience of point‐of‐care testing for influenza in Scotland in 2017/18 and 2018/19—no gain without pain. EuroSurveillance. 2020;25(44). doi:10.2807/1560-7917.ES.2020.25.44.1900419 PMC7645975 33153519

[12] Gren LH , Porucznik CA , Joy EA , Lyon JL , Staes CJ , Alder SC . Point‐of‐care testing as an influenza surveillance tool: methodology and lessons learned from implementation. Influenza Res Treat. 2013;2013:242970. doi:10.1155/2013/242970 23691297 PMC3649292

[13] Youngs J , Marshall B , Farragher M , et al. Implementation of influenza point‐of‐care testing and patient cohorting during a high‐incidence season: a retrospective analysis of impact on infection prevention and control and clinical outcomes. J Hosp Infect. 2019;101(3):276‐284. doi:10.1016/j.jhin.2018.11.010 30471317

[14] Brendish NJ , Schiff HF , Clark TW . Point‐of‐care testing for respiratory viruses in adults: the current landscape and future potential. J Infect. 2015;71(5):501‐510. doi:10.1016/j.jinf.2015.07.008 26215335 PMC7172689

[15] Clark TW , Beard KR , Brendish NJ , et al. Clinical impact of a routine, molecular, point‐of‐care, test‐and‐treat strategy for influenza in adults admitted to hospital (FluPOC): a multicentre, open‐label, randomised controlled trial. Lancet Respir Med. 2021;9(4):419‐429. doi:10.1016/S2213-2600(20)30469-0 33285143 PMC9764870

[16] Public Health England . Sources of UK flu data: influenza surveillance in the UK 2019 [Available from: https://www.gov.uk/guidance/sources-of-uk-flu-data-influenza-surveillance-in-the-uk#disease-severity-and-mortality-data

